# Navigating HER2-Low Testing in Invasive Breast Cancer: Update Recommendations for Pathologists

**DOI:** 10.3390/jpm14050467

**Published:** 2024-04-28

**Authors:** Leticia Bornstein-Quevedo, Jazmín de Anda-González, Cesar Octavio Lara-Torres, Juan Pablo Flores-Gutiérrez, Rita Dorantes-Heredia, Verónica Bautista-Piña, Perla Zaragoza-Vargas, Aldo Alcaraz-Wong, Ana Karen Soto-Sañudo, Saulo Mendoza-Ramírez, Moisés Salamanca-García, Georgina Loyola-Rodríguez, Gabriela Sofia Gómez-Macías, Mario Murguia-Perez, Marcela De Luna-Sánchez, Ricardo Villalobos-Valencia, Enrique Talamantes, Claudia Arce-Salinas

**Affiliations:** 1 Department of Immunohistochemistry and Molecular Pathology, InmunoQ, Mexico City 03200, Mexico; 2 Department of Pathology, Hospital de Oncología CMN Siglo XXI, Instituto Mexicano del Seguro Social, Mexico City 06720, Mexico; deandago@imss.gob.mx; 3 Department of Pathology, Instituto Nacional de Cancerología, Mexico City 14000, Mexico; c_laratorres@yahoo.com.mx; 4 Department of Pathology, University Hospital Dr. José Eleuterio González, Monterrey 64460, Mexico; jufloresmx@hotmail.com (J.P.F.-G.); dra.gabrielagomez@tecsalud.mx (G.S.G.-M.); 5 Department of Pathology, Hospital Médica Sur, Mexico City 14050, Mexico; rdorantesh@medicasur.org.mx; 6 Department of Pathology, Fundación de Cáncer de Mama, Mexico City 04980, Mexico; veronica.bautista@fucam.org.mx; 7 Department of Pathology, Hospital de Gineco-Obstetricia, Instituto Mexicano del Seguro Social, Mexico City 64000, Mexico; perla.zaragoza@imss.gob.mx; 8 Department of Pathology, Centro Médico Nacional de Occidente, Instituto Mexicano del Seguro Social, Guadalajara 44340, Mexico; aldo.alcaraz@imss.gob.mx; 9 Department of Pathology, Hospital Regional “Dr. Manuel Cárdenas de la Vega”, Instituto de Seguridad y Servicios Sociales de los Trabajadores del Estado, Culiacán Rosales 80230, Mexico; ana.sotos@issste.gob.mx; 10 Department of Pathology, Hospital General de México, Mexico City 06720, Mexico; dr.saulomr.oncopato@gmail.com; 11 Department of Pathology, Centro Médico Nacional 20 de Noviembre, Instituto de Seguridad y Servicios Sociales de los Trabajadores del Estado, Mexico City 03229, Mexico; moises.salamanca@issste.gob.mx; 12 Department of Pathology, Instituto de Seguridad y Servicios Sociales de los Trabajadores al Servicio de los Poderes del Estado de Puebla, Puebla 72550, Mexico; georgina.loyo47@anahuac.mx; 13 Department of Pathology, Centro Médico Nacional del Bajío, Instituto Mexicano del Seguro Social, León 37320, Mexico; mario.murguia@imss.gob.mx; 14 Department of Pathology, Hospital Central Militar, Mexico City 11600, Mexico; mapat_dlun@hotmail.com; 15 Department of Oncology, Hospital de Oncología CMN Siglo XXI, Instituto Mexicano del Seguro Social, Mexico City 06720, Mexico; ricardo.villalobos@imss.gob.mx; 16 Department of Oncology, Centro Médico Nacional La Raza, Instituto Mexicano del Seguro Social, Mexico City 02990, Mexico; enrique.talamantes@imss.gob.mx; 17 Department of Oncology, Instituto Nacional de Cancerología, Mexico City 14000, Mexico; carces@incan.edu.mx

**Keywords:** breast cancer, metastatic disease, HER2, HER2-low testing, IHC assays, ISH, HER2-targeted therapy, trastuzumab deruxtecan

## Abstract

The article discusses the importance of accurately distinguishing HER2-low from HER2-negative breast cancer, as novel ADCs have demonstrated activity in a large population of patients with HER2-low-expressing BC. While current guidelines recommend a dichotomous classification of HER2 as either positive or negative, the emergence of the HER2-low concept calls for standardization of HER2 testing in breast cancer, using currently available assays to better discriminate HER2 levels. This review covers the evolution and latest updates of the ASCO/CAP guidelines relevant to this important biomarker in breast cancer, including still-evolving concepts such as HER2 low, HER2 heterogeneity, and HER2 evolution. Our group presents the latest Mexican recommendations for HER2 status evaluation in breast cancer, considering the ASCO/CAP guidelines and introducing the HER2-low concept. In the era of personalized medicine, accurate HER2 status assessment remains one of the most important biomarkers in breast cancer, and the commitment of Mexican pathologists to theragnostic biomarker quality is crucial for providing the most efficient care in oncology.

## 1. Introduction

The human epidermal growth factor receptor 2 (HER2/neu or HER2) gene is located on the long arm of chromosome 17 and encodes the transmembrane receptor protein HER2, which has tyrosine kinase activity [1]. HER2 belongs to the epidermal growth factor receptor (EGFR) family, also known as the HER family. This family includes four members (HER1 to HER4) and, under physiological conditions, is involved in intercellular and cell-stroma communication. However, HER receptors exhibit abnormal signaling activity in a wide range of tumors. Within this family, HER2 is particularly oncogenic. HER2 has been considered a therapeutic target because HER2 gene alterations induce a malignant phenotype, it is overexpressed in 15–18% of breast cancer, and it is associated with a poor prognosis for these patients [2].

HER2 is an important prognostic and predictive biomarker in primary or metastatic breast cancer (BC). These patients should be tested for HER2 by immunohistochemistry (IHC) and/or amplification by in situ hybridization (ISH) [3] at the tumor to guide clinical treatment. Currently, breast carcinomas are classified as HER2-positive when HER2 expression is 3+ by IHC or 2+ with HER2 gene amplification by ISH. In contrast, BC with an IHC HER2 score of 0 or 1+ or an IHC score of 2+ without gene amplification are considered HER2-negative, and these tumors lack a therapeutic benefit from anti-HER2 agents. Patients with HER2-positive tumors can receive drugs that block the HER2 pathway, such as anti-HER2 monoclonal antibodies (trastuzumab, pertuzumab, and margetuximab), antibody–drug conjugates (ADC), such as trastuzumab emtansine (T-DM1) and trastuzumab deruxtecan (T-Dxd), and tyrosine kinase inhibitors (tucatinib, lapatinib, and neratinib). These drugs have drastically improved the clinical outcomes of HER2-positive BC. Recently, promising results have been reported in clinical trials for the treatment of HER2-negative BC with anti-HER2 ADC drugs. Based on these results, the concept of HER2-low in BC was proposed for the first time in 2020. This term refers to breast cancer with an IHC HER2 score of 1+ or 2+/ISH negative [4].

The identification of HER2-positive breast cancer has revolutionized the treatment of this disease in recent decades. However, despite advances, there are still significant challenges in the diagnosis and treatment of HER2-positive breast cancer, especially in developing countries such as Mexico. Therefore, it is essential to have a national consensus that brings together the experience and knowledge of experts to propose recommendations for the interpretation of HER2 in breast cancer in Mexico.

## 2. Evolution of HER2 Interpretation Guidelines

The guidelines aim to provide a detailed and comprehensive description of how to interpret HER2 test results in breast cancer patients. They focus on providing clear and detailed information about how the test is performed, what the results mean, and how they should be interpreted based on the stage of cancer and other relevant factors. Issues related to the use of different testing techniques, result interpretation, and updating HER2 guidelines in clinical practice are also addressed.

In diagnostic practice, HER2 status must be evaluated in breast cancer patients, whether at initial diagnosis, recurrence, or metastasis. This status is assigned based on the recommendations of an international group of experts. The American Society of Clinical Oncology/College of American Pathologists (ASCO/CAP) has developed recommendations for HER2 testing since 2007 [5], also taking care to include optimal pre-analytical and analytical requirements for the performance and interpretation of HER2 tests using immunohistochemistry (IHC) or in situ hybridization (ISH). The pre-analytical phase has a significant impact on the correct performance of both IHC and ISH.

It is important to emphasize the use of neutral pH (7.0) 10% buffered formalin for tissue fixation in a ratio of 1:10 of the biopsy tissue volume [6]. Adequate control of cold ischemia time is paramount to ensure the preservation of antigens, DNA, and RNA in tissues fixed in formalin and embedded in paraffin, as well as optimal fixation time (6–72 h).

In initial clinical trials, a HER2 score of 3+ by IHC or a score of 2+ with a positive fluorescent in situ hybridization (FISH) test (defined by a HER2/CEP17 ratio ≥ 2 in >50% of neoplastic cells) was used as eligibility criteria. In these trials, an IHC score of 3+ was defined as intense/strong and complete membrane staining in >10% of neoplastic cells, and 2+ as complete circumferential membrane staining of weak to moderate intensity in >10% of neoplastic cells [6]. In the ASCO/CAP 2007 guidelines, the positivity threshold was raised to >30% of neoplastic cells by IHC and HER2/CEP17 ≥ 2.2 by ISH in order to reduce the number of false positives.

In 2009, ASCO/CAP published a supplement to the 2007 guidelines regarding HER2 heterogeneity in ISH testing. HER2 genetic heterogeneity was defined as the presence of ≥5% to <50% of invasive tumor cells with a ratio ≥ 2.2 when using dual probes or ≥6 HER2 signals/cell using single probes. The recommendation was to review all neoplastic tissue to identify heterogeneity and evaluate two to four representative fields of invasive carcinoma. Groups (>20 cells) with HER2 amplification by ISH should be evaluated separately for HER2/CEP17 and/or HER2 signals/cell [7].

The ASCO/CAP 2013 guidelines [8] reverted the positivity threshold to the original >10% of neoplastic cells by IHC and HER2/CEP17 ratio ≥ 2 by ISH. These recommendations aimed to avoid false negative results, which could deny potentially useful treatment (anti-HER2 therapy) to breast cancer patients.

The guidelines introduced the concept of an ISH algorithm, which represents a two-step approach in the evaluation of results, taking into account the HER2/CEP17 ratio, followed by the analysis of the average number of HER2 copies when the HER2/CEP17 ratio is <2. This algorithm helped to avoid misclassification of HER2 amplification in cases with an abnormal number of copies of the centromeric region of chromosome 17 (CEP17) (monosomy or polysomy of chromosome 17) [8]. Finally, the ASCO/CAP 2013 recommendations also address the issue of HER2 heterogeneity [8], which was defined as a separate population of HER2-positive or ISH-positive tumor cells representing at least 10% of the entire neoplastic cell population.

In 2018, an update to the guidelines was published with a focus on five groups for interpreting ISH [3]. Groups 1 (HER2/CEP17 ratio ≥ 2, HER2 average copy number/nucleus > 4) and 5 (HER2/CEP17 ratio < 2, HER2 average copy number/nucleus < 4) represent the two extremes of the HER2 evaluation spectrum (presence and absence of HER2 amplification, respectively) and account for 95% of ISH test results. The reproducibility of these tests can be affected by various pre-analytical and analytical issues. Formalin fixation and technical and biological artifacts are factors that significantly affect the analytical reliability of IHC studies, which complicates the identification of low HER2 expression in terms of both false positives and false negatives [9].

Between 5% and 15% of cases are classified into groups 2, 3, and 4, which present challenging interpretation scenarios characterized by the presence of monosomy, polysomy, or tumor heterogeneity [10]. Studies available to date on the impact of the 2018 guidelines indicate that their application leads to an increase in the number of negative HER2 tests. This observation stems from the reclassification of cases in groups 2 and 4, which are recommended to be categorized as negative. It is important to note that ISH testing should be available to properly classify equivocal cases (2+).

The initiative to conduct a new review of the ASCO-CAP guidelines emerged following the publication of the clinical study DESTINY-Breast04 in 2022. This study documented a significant improvement in survival among patients with breast cancer without HER2 overexpression or amplification but with IHC scores of 1+ or 2+ and non-amplified results on ISH who were treated with trastuzumab deruxtecan [11].

In this context, the current ASCO/CAP 2023 guidelines focus on recognizing a new indication for trastuzumab deruxtecan when HER2 is neither overexpressed nor amplified but shows an IHC score of 1+ or 2+ without amplification by in situ hybridization. This eligibility does not apply to IHC 0. The guidelines indicate that, although it is premature to create new categories for HER2 expression outcomes (e.g., HER2-low), it is clinically relevant to establish best practices for distinguishing between IHC 0 and 1+. This update reaffirms previous recommendations regarding HER2 reporting and provides new insights on how HER2 results should be reported, emphasizing the current relevance of distinguishing between IHC 0 and 1+ [11].

### Recommendations

The guidelines from previous ASCO-CAP HER2 testing updates in 2013 and 2018 are reaffirmed for conventional anti-HER2 therapies targeting HER2 signaling pathways. While no alterations have been introduced to the existing recommendations, it is important to acknowledge that, in the case of metastatic patients lacking HER2 overexpression or gene amplification, an IHC 1+ or 2+ outcome may render patients suitable for treatment focusing on non-amplified/non-overexpressed levels of HER2 expression. This eligibility does not extend to cases with an IHC 0 result. Trastuzumab deruxtecan currently stands as the sole available agent targeting such non-amplified/non-overexpressed levels of HER2 expression.

According to the ASCO/CAP guidelines, a score of 3+ on IHC indicates positivity; an IHC score of 2+ can also indicate positivity if there is ISH evidence of HER2 gene amplification in the form of a HER2/CEP17 ratio of 2.0 or higher, and/or a HER2 copy number of 6.0 or higher.

## 3. HER2-Low Breast Cancer

Traditionally, breast carcinomas have been classified as HER2-positive and HER2-negative. This dichotomous diagnosis, proposed by the ASCO/CAP update of 2018 [3], considers HER2-positive BC (HER3+/ISH amplified) to represent 15% of cases and the rest (85%) to be HER2-negative. A BC with an IHC HER2 score of 2+ and no evidence of HER2 gene amplification (IHC 2+/ISH−) is currently classified as HER2-negative, similar to tumors with an IHC score of 0 or 1+. These patients do not benefit from conventional anti-HER2 therapy.

A new category of breast cancer patients with reduced HER2 expression has recently been identified, known as HER2-low, defined as IHC 1+ or IHC 2+ ISH non-amplified, which represents 45–64% of BC (4) [12]. The majority show invasive ductal phenotype, histological grades 1 and 2, estrogen receptor-positive (ranges: 43.5–67.6%), progesterone receptor positive (approximately 80%), luminal molecular subtype, and clinically stage II [13].

There are several staining patterns of HER2 protein expression, including the combination of staining intensity (faint, weak, moderate, and strong), membrane circumferential (complete vs. incomplete), and cutoff point (e.g., 10%) to classify the percentage of HER2 staining in invasive tumor cells. According to the ASCO/CAP guidelines [3], HER2 IHC scoring is defined as follows: HER2 0+ indicates either no observed staining or incomplete membrane staining that is faint/barely perceptible in <10% of invasive tumor cells, HER2 1+ indicates incomplete faint perceptible membrane staining within >10% of invasive tumor cells, HER2 2+ indicates weak to moderate complete membrane staining observed in >10% of invasive tumor cells, and HER2 3+ indicates complete, intense circumferential membranous staining in >10% of invasive tumor cells.

HER2 staining patterns are categorized as homogenous or heterogeneous. A homogenous pattern signifies an evenly distributed HER2 staining throughout the tumor, while a heterogeneous pattern indicates geographic variations in HER2 staining within the same tumor. Heterogeneous patterns further include clustered (regional) patterns, characterized by segregated populations of HER2-stained and non-stained tumor cells, mosaic (intermixed) patterns, where HER2-stained cells are intermixed with a non-stained tumor cell, and scattered patterns, where isolated HER2-stained cells are observed amidst a background of non-stained tumor cell population [9].

The identification of HER2-low is relatively straightforward for those with an IHC score of 2+, which is negative for ISH. These tumors, which show recognizable levels of protein expression that are not sufficient to score 3+, are identified as part of the existing and well-established HER2 testing protocols to define HER2 positivity. However, the lower limit of protein expression required for response to ADCs is not yet clearly defined. The clinical trial of anti-HER2 ADC used the existing ASCO/CAP criteria [3] to define 1+ and distinguished it from tumors with an IHC score of 0 [14]. There are several staining patterns of HER2 protein expression, including the combination of staining intensity (faint, weak, moderate, and strong), membrane circumferential (complete vs. incomplete), and cutoff point (e.g., 10%) to classify the percentage of HER2 staining in invasive tumor cells. Despite the overall high concordance in the classification of HER2-positive and negative tumors, the concordance in distinguishing neoplasms with IHC scores of 1+ and 0 using existing criteria remains low [15,16,17].

The intratumoral heterogeneity of HER2 is observed in a small group of tumors and has significant clinical consequences [17]. This heterogeneity is more common in breast tumors that have equivocal HER2 expression (IHC 2+), which accounts for approximately 10% of cases and has low HER2 gene amplification. This phenomenon can lead to a misdiagnosis of the HER2 status [18,19]. HER2 staining patterns by IHC can be either homogeneous or heterogeneous. The homogeneous pattern corresponds to the uniform distribution of neoplastic cells. Intratumoral genetic heterogeneity has been well-documented in several types of neoplasms, including breast cancer. It is well known by pathologists who systematically evaluate HER2 in diagnostic practice that overexpression can present different heterogeneous patterns [7,20,21]. Three different types of HER2 heterogeneous cell distribution have been described: clustered, mosaic, and scattered [22]. The clustered or clonal type shows two topographically distinct tumor cell clones, one with HER2 amplification and the other with a normal HER2 state. The mosaic type is the most common and presents a diffuse mixture of cells with different HER2 protein expressions and HER2 gene copy numbers. The scattered type shows isolated HER2-amplified cells in a predominantly HER2-negative tumor cell population. These isolated HER2-positive cells often have low HER2 amplification levels and have a limited response to anti-HER2 therapy compared to the clustered type [23].

The expression of HER2-low has been shown to be highly dynamic over time, with a significant portion of HER2-low tumors transitioning to HER2-0 and vice versa, either in residual disease following neoadjuvant therapy [13] or after tumor relapse [13,14].

This dynamism is likely due to multiple factors; HER2 expression can be modulated by various stimuli within the tumor microenvironment, as well as the impact of prior treatment [15,16]. Other potential factors include pre-analytical and analytical challenges in HER2 testing methods, leading to high discordance in assessment [17,18]. Irrespective of the involved factors, this observation emphasizes the need to re-evaluate the HER2 status during the patient’s disease, even when the tumor was HER2-0 in a prior biopsy, potentially enabling access to T-Dxd treatment in case of a shift to HER2-low expression. It is noteworthy that in the DESTINY-Breast04 trial, both archival and fresh tumor biopsy samples were accepted, and the presence of previous HER2-0 samples was not an exclusion criterion [19]. The appropriate timing for defining HER2-low is yet to be elucidated. However, even patients whose latest biopsy showed HER2-0 results might be considered for T-Dxd treatment if they exhibited HER2-low scoring in any prior biopsy.

Immunohistochemistry (IHC) is a semi-quantitative test with both advantages and limitations. Pre-analytical, analytical, and post-analytical variables, as well as inter- and intra-observer variability [14] and the use of different antibodies, can all affect the interpretation of HER2, especially in the case of HER2 low status. In 2022, the FDA approved the rabbit monoclonal primary antibody VENTANA PATHWAY anti-HER2/neu (4B5) as the sole diagnostic test to identify patients with metastatic breast cancer with low HER2 expression for whom T-Dxd may be considered as a specific targeted treatment [24,25]. For tumors that exhibit incomplete membrane reactivity with strong or moderate intensity (excluding very focal areas where the majority is evidently 3+), reflex ISH testing should be conducted. It is important to mention that there is no direct link between the HER2-low category and ISH ratios or copy numbers. Therefore, laboratories that rely on ISH as their primary screening method instead of the two-tiered approach may not be able to detect cases falling under the HER2-low category.

**Figure 1:**
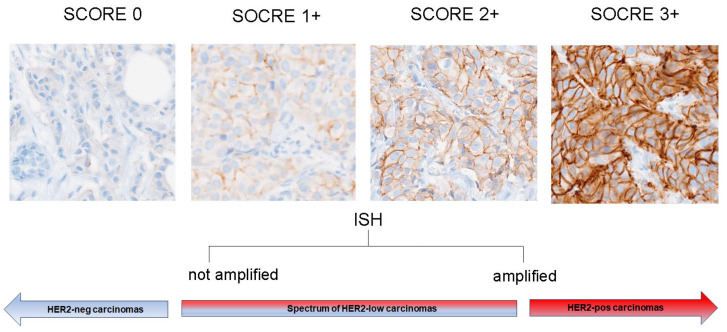
Definition of HER2 status in breast cancer within the context of the HER2-low category.

Currently, HER2 testing is used as a companion diagnostic in clinical practice. We believe that the introduction of the HER2-low concept will not require a modification in testing protocols. Although we consider that there should be an improvement in the scoring and reporting criteria, the 2023 ASCO/CAP guidelines affirm the categorization of HER2 IHC 1+ or 0 results as HER2-negative, indicating the absence of HER2 overexpression, in accordance with the existing scoring criteria (See Figure 1). Ensuring the inclusion of the semi-quantitative IHC score in reports is of paramount importance to effectively identify patients eligible for trastuzumab deruxtecan treatment. For instance, as an illustration, “HER2-negative for protein overexpression (1+ staining present)” [26].

### Recommendations

While it is premature to alter the terminology for reports on low levels of IHC expression in HER2 (e.g., HER2-Low), pathology laboratories should include a footnote in their HER2 test reports (both IHC and ISH) with the following recommended comment:

“Patients with breast cancer having HER2 IHC 3+ or IHC 2+/ISH amplified may be eligible for various therapies targeting HER2 signaling pathways. Invasive breast cancers yielding ‘HER2 negative’ results (IHC 0, 1+, or 2+/ISH non-amplified) are more specifically considered ‘HER2 negative for protein overexpression/genetic amplification’, as non-overexpressed levels of the HER2 protein may be present in these cases. Patients with breast cancer exhibiting HER2 IHC 1+ or IHC 2+/ISH non-amplified may be eligible for targeted treatment with cytotoxic drugs (IHC 0 is not an eligibility criterion)” [11].

Given that eligibility for trastuzumab deruxtecan (IHC 1+ or IHC 2+/ISH non-amplified) may depend on the IHC 0/IHC 1+ threshold (although the clinical validity of this threshold has not yet been proven), pathologists may undertake their best practice efforts to distinguish IHC 1+ results from 0 through the following practices:

1. Examine IHC-HER2 stained slides using the scoring criteria from the standardized ASCO-CAP guidelines;
2. Evaluate IHC-HER2 at high power (40×) to discriminate between staining 0 and 1+;
3. Consider a second pathologist review when results are close to the interpretative threshold of 0 versus 1+ (>10% of cells with weak/barely perceptible incomplete membrane staining);
4. Use controls with a protein expression range (including 1+) to help ensure the assay has an appropriate detection limit;
5. Consider the preanalytical conditions of tissue samples from both primary and metastatic sites in breast cancer.

## 4. Clinical Considerations for Patients with HER2-Low BC

From a clinical standpoint, low HER2 breast cancer appears to be more common in older patients and men with breast cancer, and it also shows a greater involvement of axillary lymph nodes compared to HER2 0 disease [22].

Trastuzumab deruxtecan (T-Dxd) is the second HER2-directed antibody–drug conjugate (ADC) approved by the FDA for HER2-positive metastatic breast cancer and the first agent directed towards HER2 for inoperable or metastatic low HER2 breast cancer [27]. T-Dxd consists of an anti-HER2 immunoglobulin G1 antibody, a cleavable tetrapeptide linker, and a membrane-permeable topoisomerase I inhibitory with a drug–antibody ratio of 8:1 [19,28].

In the DESTINY Breast-04 trial [19], T-Dxd was evaluated in 557 patients (494 hormone receptor [HR]-positive and 63 triple-negative breast cancer [TNBC]) with inoperable or metastatic low HER2 breast cancer who had received one or two prior lines of chemotherapy. Treatment with T-Dxd (5.4 mg/kg every 3 weeks), in addition to physician’s choice chemotherapy, resulted in a confirmed objective response rate of 52.6% in HR-positive patients and 52.3% in the overall study population, compared to physician’s choice chemotherapy (16.3%). Compared to physician’s choice chemotherapy, T-Dxd significantly improved progression-free survival (PFS) in HR-positive patients (10.1 vs. 5.4 months, hazard ratio [HR] 0.51; *p* < 0.001) and in the overall population (9.9 vs. 5.1 months, HR 0.50; *p* < 0.001). Overall survival (OS) also improved with T-Dxd treatment among HR-positive patients (23.9 vs. 17.5 months, HR 0.64, *p* = 0.003) and in the overall population (23.4 vs. 16.8 months, HR 0.64, *p* = 0.001). Similarly, in an exploratory analysis conducted on a small number of TNBC patients, T-Dxd also improved PFS (8.5 vs. 2.9 months, HR 0.46) and OS (18.2 vs. 8.3 months, HR 0.48). Unlike other anti-HER2 agents, T-Dxd’s unique clinical benefits in low HER2 BC may be associated with “indirect destruction” mechanisms due to the highly membrane-permeable payload, high drug–antibody ratio, and cleavable linker, primarily as a means of delivering antibody-conjugated drugs, rather than directly inhibiting HER2 dimerization or blocking downstream signaling.

T-Dxd is generally manageable and tolerable in terms of safety profile, with the most common adverse effects being gastrointestinal disturbances, myelotoxicity, and alopecia. Approximately 28% of patients experienced adverse reactions [13]. The most severe adverse effect is interstitial lung disease (ILD/pneumonitis) [29].

Trastuzumab duocarmazine (SYD985) is a HER2 immunoconjugate with trastuzumab and duocarmazine [30]. After binding and internalization of HER2, the drug cleaves in the lysosome and releases a toxin (DUBA), which alkylates DNA and causes cell death. It has been shown that cleavage of the drug from its anchor can also be extracellular, causing an expansive effect of cell death to surrounding cells that is not mediated by HER2 [31]. In the pivotal, multicenter, open-label, randomized phase III trial called TULIP [24] in patients with metastatic, unresectable, and pretreated breast cancer (MBC), comparing trastuzumab duocarmazine with the researcher’s choice of medical treatment, the primary results were very promising with a progression-free survival of 7.0 months for trastuzumab duocarmazine versus 4.9 months for the investigator’s chosen treatment.

Other antibody–drug conjugates, such as ALT-P7 [25] and PF-06804103 [27], have shown a PFS of six months and an objective response rate of 52.4%, respectively. The phase III study DESTINITY-Breas 06 [32] evaluates T-Dxd compared to the researcher’s choice of chemotherapy in patients with metastatic HER2-low breast cancer who have positive hormone receptors and whose disease has progressed on endocrine therapy. The results are pending. Possible resistance mechanisms associated with the use of ADCs may include loss of antibody-mediated activity, dysfunctional intracellular trafficking, and overexpression of transporters that move drugs outside the cell. Among the strategies being developed to overcome these resistance mechanisms is the synergy of ADCs in combination with immunotherapy [29]. Trastuzumab deruxtecan (T-Dxd) has been approved by the FDA for inoperable or metastatic low HER2 breast cancer. In a clinical trial, T-Dxd showed significant improvements in progression-free survival (PFS) and overall survival (OS) compared to physician’s choice chemotherapy. It is generally manageable and tolerable in terms of safety profile, with gastrointestinal disturbances, myelotoxicity, and alopecia being the most common adverse effects. Other antibody–drug conjugates, such as trastuzumab duocarmazine (SYD985), have also shown promising results. Strategies to overcome resistance mechanisms include the synergy of ADCs in combination with immunotherapy. The phase III study DESTINITY-Breas 06 is currently evaluating T-Dxd’s efficacy compared to chemotherapy.

### Recommendations

Clinicians should consider trastuzumab deruxtecan (T-Dxd) as a viable treatment option for inoperable or metastatic low HER2 breast cancer, especially in patients who have received one or two prior lines of chemotherapy. T-Dxd has shown significant improvements in progression-free survival and overall survival compared to physician’s choice chemotherapy in hormone receptor-positive and overall patient populations. However, careful monitoring for interstitial lung disease (ILD/pneumonitis), the most severe adverse effect of T-Dxd, is necessary.

Trastuzumab duocarmazine (SYD985), an immunoconjugate with trastuzumab and duocarmazie, has shown promising results in the treatment of metastatic, unresectable, and pretreated breast cancer. Future studies, including the ongoing DESTINY-Breas 06, are expected to provide further insight into the efficacy of T-Dxd.

Currently, clinicians in the field use imaging modalities or response biomarkers to assess resistance toward ADCs. Nevertheless, the comprehensive understanding of ADC resistance mechanisms remains incomplete, prompting investigations into their combination with immunotherapeutic agents for mitigation. Efforts to address resistance are actively pursued through controlled clinical trials. Illustratively, the phase 1b/2 BEGONIA trial (NCT03248492) exemplifies this approach by evaluating the synergistic effects of trastuzumab and durvalumab in patients with triple-negative breast cancer and low HER2 expression, demonstrating promising preliminary outcomes [33]. Concurrently, the ongoing NCT04042701 trial explores the efficacy of combining trastuzumab deruxtecan with pembrolizumab [34]. These investigations serve as notable instances wherein the integration of trastuzumab deruxtecan with immunotherapy is explored to overcome tumor resistance.

In HER2-negative patients, there are currently established treatment protocols primarily dependent on clinical stage and estrogen/progesterone receptor status. However, we, as authors, consider that this information falls outside the scope of this article.

## 5. Conclusions

The ASCO/CAP guidelines describe how to interpret HER2 test results in breast cancer patients. The guidelines cover different testing techniques, results interpretation, and updating of HER2 guidelines in clinical practice. To ensure accurate test results, it is crucial to follow the optimal pre-analytical and analytical requirements, including the use of neutral pH (7.0) 10% buffered formalin for tissue fixation and adequate control of cold ischemia time. The HER2-low category, which represents 45–64% of breast cancer cases, has been introduced as a new category of patients but does not require a modification in testing protocols.

In the current ASCO/CAP 2023 guidelines, while there are no changes to the previous recommendations, it is imperative to be mindful that for metastatic patients without HER2 overexpression or genetic amplification, an IHC result of 1+ or 2+ may render them eligible for targeted treatment directed at non-amplified/non-overexpressed levels of HER2 (IHC 0 results do not apply to eligibility), for which trastuzumab deruxtecan is currently the sole available agent.

## Data Availability

No new data were created or analyzed in this study. Data sharing is not applicable to this article.
